# Comparing and Contrasting MERS, SARS-CoV, and SARS-CoV-2: Prevention, Transmission, Management, and Vaccine Development

**DOI:** 10.3390/pathogens9120985

**Published:** 2020-11-26

**Authors:** Mohammad Oves, Mithunan Ravindran, Mohd Ahmar Rauf, Mohammad Omaish Ansari, Maryam Zahin, Arun K. Iyer, Iqbal M. I. Ismail, Meraj A. Khan, Nades Palaniyar

**Affiliations:** 1 Centre of Excellence in Environmental Studies, King Abdulaziz University, Jeddah, Makkah 21589, Saudi Arabia; owais.micro@gmail.com (M.O.); iqbal30@hotmail.com (I.M.I.I.); 2 Program in Translational Medicine, SickKids Research Institute, The Hospital for Sick Children, Toronto, ON M5G 1X8, Canada; mithunan.ravindran@sickkids.ca; 3 Faculty of Medicine, University of Toronto, Toronto, ON M5S1A8, Canada; 4 Department of Pharmaceutical Sciences, Eugene Applebaum College of Pharmacy and Health Sciences, Wayne State University, Detroit, MI 48201, USA; hb7059@wayne.edu (M.A.R.); arun.iyer@wayne.edu (A.K.I.); 5 Center of Nanotechnology, King Abdulaziz University, Jeddah, Makkah 21589, Saudi Arabia; omaishchem@gmail.com; 6 Center for Predictive Medicine and James Graham Brown Cancer Center, University of Louisville, Louisville, KY 40202, USA; m0zahi01@louisville.edu; 7 Department of Chemistry, Faculty of Science, King Abdulaziz University, Jeddah, Makkah 21589, Saudi Arabia; 8 Department of Laboratory Medicine and Pathobiology, Faculty of Medicine, University of Toronto, Toronto, ON M5S1A8, Canada

**Keywords:** coronavirus, MERS-CoV, 2019-nCoV, SARS-CoV-2, COVID-19, phylogeny, innate immunity, lung pathogenesis, epidemiology, modeling, therapy, vaccine development

## Abstract

The COVID-19 pandemic is responsible for an unprecedented disruption to the healthcare systems and economies of countries around the world. Developing novel therapeutics and a vaccine against SARS-CoV-2 requires an understanding of the similarities and differences between the various human coronaviruses with regards to their phylogenic relationships, transmission, and management. Phylogenetic analysis indicates that humans were first infected with SARS-CoV-2 in late 2019 and the virus rapidly spread from the outbreak epicenter in Wuhan, China to various parts of the world. Multiple variants of SARS-CoV-2 have now been identified in particular regions. It is apparent that MERS, SARS-CoV, and SARS-CoV-2 present with several common symptoms including fever, cough, and dyspnea in mild cases, but can also progress to pneumonia and acute respiratory distress syndrome. Understanding the molecular steps leading to SARS-CoV-2 entry into cells and the viral replication cycle can illuminate crucial targets for testing several potential therapeutics. Genomic and structural details of SARS-CoV-2 and previous attempts to generate vaccines against SARS-CoV and MERS have provided vaccine targets to manage future outbreaks more effectively. The coordinated global response against this emerging infectious disease is unique and has helped address the need for urgent therapeutics and vaccines in a remarkably short time.

## 1. Introduction to the Clinical Manifestations of COVID-19

The current understanding of the spread of COVID-19 indicates that the outbreak started as early as 1 November 2019 [1,2,3,4,5]. Shortly thereafter, SARS-CoV-2 was identified as the virus causing a cluster of “pneumonia of unknown etiology” cases in Wuhan, China in December 2019. This prompted the WHO to declare the outbreak a Public Health Emergency of International Concern (PHEIC) on 30 January 2020 [6]. This decision was influenced by the rapid spread of the virus, as well as its ability to cause a high rate of mortality and morbidity. Outside of China, transmission initially involved travel-related cases, but many countries had reported community transmission by late February and early March. The next significant development occurred on 11 March 2020, when the WHO announced COVID-19 to be a global pandemic.

Several excellent review articles have attempted to summarize the many new discoveries about COVID-19 [7,8,9,10,11,12]. However, there is a vast amount of new information on COVID that has been published daily. According to a recent report from the United States Centers for Disease Control and Prevention (CDC) involving 370,000 patients, the initial presentation of COVID-19 involves cough (50% of patients), fever (43%), myalgia (36%), headache (34%), dyspnea (29%), and sore throat (20%) [13]. Gastrointestinal manifestations of COVID-19 are now well-established and involve diarrhea (19% of patients), nausea and vomiting (12%), and abdominal pain (<10%) [13]. Additionally, neurological manifestations of COVID-19 have been widely reported, with some estimates as high as 36.4% of patients suffering from a variety of symptoms, including anosmia, delirium, seizures, and acute cerebrovascular disease [14]. Although more rare, recent reports indicate that younger COVID-19 patients may have skin lesions, blood clots, and strokes, though the numbers vary considerably depending on the information [15,16,17,18,19,20,21].

Furthermore, 81% of patients present with mild cases (involving mild or no pneumonia), 14% develop severe disease (pneumonia with hypoxia and dyspnea), and 5% have a critical illness with respiratory failure and multi-organ failure requiring admission to the ICU [15,22,23]. Why the clinical manifestations of COVID-19 can be so different even in patients with similar demographic characteristics and comorbidities remain an important question. Several theories have been proposed including varying levels of ACE2 expression, the receptor target of SARS-CoV-2, as well as differences in the respiratory microbiome, which may affect the response of the host immune system to the virus [24]. Patients with mild symptoms are expected to recover in about two weeks, while more severe cases will take around 3 to 6 weeks [18]. This long recovery period has significant implications for ICU bed capacity, which has been stretched to the limit in hospitals around the world. While COVID-19 can present with pneumonia, the most lethal complication is acute respiratory distress syndrome [25]. Among patients admitted to the hospital due to COVID-19, 41.8% develop acute respiratory distress syndrome (ARDS) with 52.4% mortality [26,27].

In this article, we examine the origin of the virus, its spread to various countries, the phylogeny of the evolving virus, real-time monitoring of the spread of the disease through computer modeling approaches to help treat infections, and vaccines to limit future SARS-CoV-2 outbreaks.

## 2. Origin, Detection, and Transmission of SARS-CoV-2

Origin: Coronavirus was first detected in chickens in the 1930s and eventually in humans’ nasal cavities suffering from the common cold in the 1960s [28]. The first human coronavirus was isolated by different techniques in the UK and the United States. In the UK, the Common Cold Unit identified the first known coronavirus infecting humans in sample B814 collected from a patient suffering from the common cold [28,29,30]. In this sample, the viral agent was visualized by lysing infected cells that were a part of the organ culture. Under electron microscopy, spike proteins present on the virus appeared as a halo or diamond-like projections on a crown, hence, the virus was named “coronavirus.” The researchers found that the colds produced by this virus were distinct from rhinoviruses, in that coronavirus required longer incubation periods and induced less productive cough. This discovery was independently verified in the United States in 1962, through samples taken from medical students with the common cold and cultured in human kidney cells [31]. The initial prototype strain in this investigation was designated as 229E, which would go on to be the name of the first coronavirus capable of infecting humans. Six more coronaviruses have since been identified. Similar to 229E, HKU1, NL63, and OC43, which usually cause mild to moderate upper-respiratory tract illnesses, SARS-CoV, MERS-CoV, and SARS-CoV2 can cause severe disease [32,33,34,35]. Previous coronaviral epidemics caused by SARS-CoV (10%) and MERS-CoV (30%) resulted in higher mortality rates than the current SARS-CoV-2 (~5%); however, all three of these viruses induce a lower airway pathological response [34,35,36,37]. SARS-COV and MERS-COV primarily infect alveolar type II cells, which secrete pulmonary surfactant and innate immune antiviral defense proteins, but use angiotensin-converting enzyme 2 (ACE2) and dipeptidyl peptidase 4 (DPP4) surface receptors, respectively [37,38,39,40,41].

Viral detection: Given that SARS-CoV-2 is an RNA virus, current diagnostic testing involves real-time reverse transcriptase-polymerase chain reaction (rRT-PCR) [42]. Current protocols recommend first using an E gene probe that is capable of detecting both bat and human coronaviruses [43]. This is then confirmed using RNA-dependent RNA polymerase (RdRp) probes, one that detects both bat and human coronaviruses, and one that is specific to SARS-CoV-2 [43]. Probes for the N gene were found to be not as analytically sensitive [43]. Generally, rRT-PCR is considered to be highly sensitive and highly specific; however, it is important to consider the role of false-negatives, especially in the context of improper data collection. This would most likely happen due to limitations in the number of healthcare staff and the availability of personal protective equipment. As reported by Wang et al., 2020, the detection rate varies depending on where the sample is taken [44]. In patients positive for COVID-19, bronchoalveolar lavage (BAL) had the highest positive rate of 93%, followed by 72% in sputum, 63% for nasal swabs, 32% for pharyngeal swabs, 29% in feces, and 1% in the blood [44,45]. This pattern supports a respiratory route of transmission, which has been correlated clinically. This pattern is also consistent with a severe infection of the lower airways. Given that SARS-CoV-2 has also been detected in patient stool samples, there is a possibility of fomite spread through contaminated surfaces, especially in a nosocomial context [45,46].

**Figure 1:**
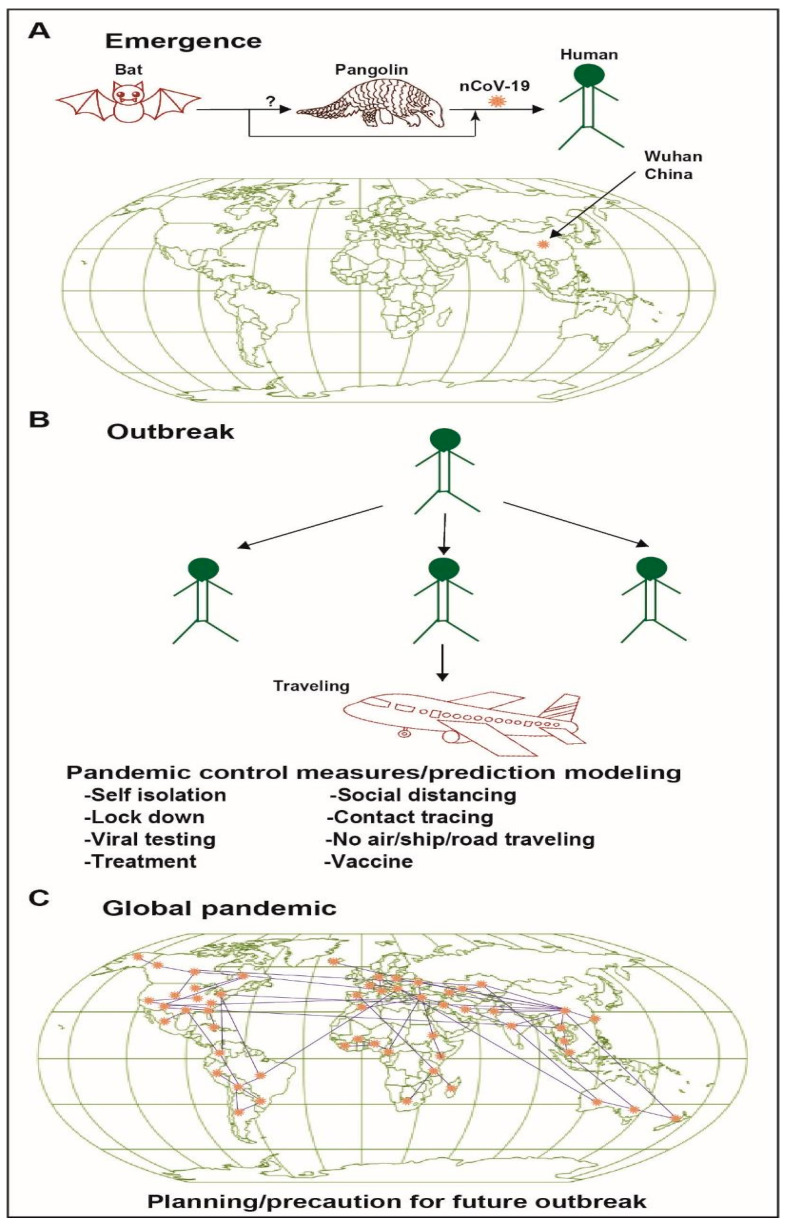
A graphical overview of the emergence and spread of SARs-CoV-2. (**A**) Shows the origin of the virus from a bat and or pangolin spreading to human. (**B**) Human to human transmission was much contributed by air travel, and community spread. (**C**) Mostly international air or interstate traveling caused the global pandemic.

Viral transmission: Human-to-human transmission of SARS-CoV-2 is now well established with an R0 ranging from 1.94 to 2.68, indicating the challenges in accurately determining this value [47]. The R0 value is difficult to estimate due to limitations in testing availability from country to country. This coronavirus is transmitted mainly by infectious secretions via the respiratory route and direct inhalation of droplets as well as through fomite spread. The mean incubation period for SARS-CoV-2 is 5.1 days, with 97.5% of patients showing symptoms by 11.5 days and 99% by 14 days [38,48]. The role of asymptomatic carriers has not yet been modeled in a meaningful way, largely due to limited testing capacity, and likely is contributing substantially to the spread of SARS-CoV-2 [49]. From the epicenter of the outbreak, the virus has now spread to the rest of the world (Figure 1).

## 3. Predicting the Spread of SARS-CoV-2 Through Phylogenetic Analysis and Computer Modeling

**Figure 2:**
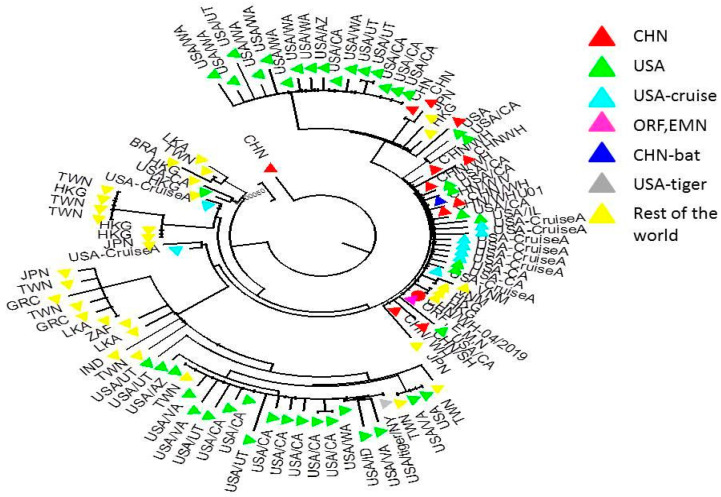
Phylogenetic tree showing the relationship among SARS-CoV-2 isolated from various locations at different time points of the pandemic. The genomic sequences of SARS-CoV-2 (MN908947.3) and all the other full genome sequences deposited to Genbank were identified (BLAST RID BBFPCRN016), and used for constructing the phylogenetic tree (MEGA 7).

Numerous viral sequences have been collected from patient airway samples and are continuously being deposited to the public domains with little or no time delay. These deposited sequences provided valuable real-time opportunities for identifying the emergence of a mutant virus and tracking the spread of the virus globally [1,2,3,4,5,50,51,52,53]. Phylogenetic analyses of available full-length sequences show that the virus has spread rapidly to different cities and countries while mutating its genome to various degrees (~100 countries; Figure 2). The phylogenetic tree also shows that the SARS-CoV-2 virus was first identified in Wuhan, China and spread to many countries around the world. This will have mainly occurred dictated by patterns in international travel, which we will discuss in greater detail, including how this form of modeling can help predict future transmission. Although the virus detected had a close ancestral relationship to the Wuhan isolate in other countries, no significant spread was reported. These differences could imply that specific versions of the virus may have the differential ability to spread within a given population or reflect the public health measures taken in those countries.

With the benefit of improved technology, viral sequences are being continuously collected from patient airway samples and being deposited to public domains with little or no time delay. These deposited sequences provide valuable real-time opportunities for identifying the emergence of viral mutations and tracking the spread of the virus globally. Investigating sequence similarity through phylogenetic analysis has shown some promise in characterizing the source of the outbreak and predicting how it might spread. Through analyzing the topology of a phylogenetic tree, Sun et al., (2020) showed that the virus likely originated in Wuhan with a highly concentrated, if not single, human transmission event [52]. According to their analysis, this interspecies transmission would have happened recently because of the high degree of similarity between samples, which would otherwise be unusual for a rapidly spreading RNA virus [52]. Wang et al., (2020) used a similar analysis to trace the source of infection for a single patient with international travel and, therefore, multiple exposures, a process that could be scaled up [53]. Finally, Forster et al., (2020) used character-based phylogenetic networks to posit that there are three central variants of the virus A, B, and C [2]. Interestingly, they found that variants A and C were more common in Europe and America, while B was more common in East Asia, which points to many areas of future research as to why this might be the case [2,3,53].

Especially in the early days of the current outbreak, prior to it being classified as a pandemic, concrete data regarding the number of cases and patterns in the transmission of the virus necessary to inform public health decision making was scarce. In this context and even once concrete data became available, large-scale computer modeling is a critical element of managing the spread of SARS-CoV-2. While many groups have been working to develop prediction models, we will highlight the underlying considerations behind two such models. The first major model, developed by Brockmann et al., (2013), uses a network-driven contagion [51]. This model replaces the conventional geographic distance with “effective distance,” which is determined by traffic between two points based on an underlying mobility network. In this case, Brockman et al. used the worldwide air transportation network (WAN), which consists of 3893 airports serving as nodes and 51,476 flight routes serving as directed links, as the underlying mobility network. Using the WAN, this model can generate shorter distances between two points with higher traffic and vice-versa. This model also enables the calculation of relative import risk, which estimates what proportion of individuals leaving the initial airport A by plane will arrive at a given airport B. Together, this information enables modeling of the most probable spreading routes. At the time the model was first published in 2013, it was successfully applied to the 2003 SARS epidemic and 2009 H1N1 pandemic. Ultimately, it was found that the predictions of this model could also be successfully applied to the current SARS-CoV-2 outbreak. Given that early in the pandemic, information such as R_0_ was not reliably available, this model was able to provide valuable and accurate predictions of disease spread that would be able to inform public health decisions before the availability of concrete data [51].

Another recent model developed by researchers in Hong Kong went a step further by incorporating not only air traffic data but also individual mobility through tracking of location-based interactions from the database of Tencent, the company which owns WeChat, among other services. This data was used in combination with data from the Chinese Centre for Disease Control on the export of COVID-19 cases from Wuhan, China, to cities outside of China. The resulting model was able to accurately predict the spread of COVID-19 in other cities within China, again enabling public health decision making. Crucially, such models can provide real-time updates on parameters such as R_0_, incubation time, mortality rate, etc., which is not possible with static data sets in the published literature [54,55].

## 4. Pathogenesis of COVID-19: Hijacking Cells, Viral Replication, and Alveolar Damage

SARS-CoV-2, like other coronaviruses, is a non-segmented positive-sense RNA virus, approximately 29.9 kb in length. The virus consists of four main structural proteins, namely three envelope proteins spike (S), membrane protein (M), and envelope protein (E), in addition to the nucleocapsid protein (N) [56,57]. S protein creates the characteristic crown appearance and mediates the entry of the virus into cells. The cellular target for the S protein is the transmembrane protein ACE2 [40,42,58]. ACE2 is found in the lungs, the walls of arteries, the heart, and kidneys and has a physiologically antagonistic effect on ACE, which functions to increase blood pressure through facilitating the renin-angiotensin-aldosterone system (RAAS) [40,58,59]. ACE2 antagonizes the RAAS system by reducing the quantity of angiotensin I and angiotensin II but also by converting angiotensin II to angiotensin 1–7, which is known to have a bradykinin-mediated vasodilation effect [60,61]. It has been shown that the S protein on SARS-CoV-2 is 10–20 times more likely to bind to ACE2 than the other S proteins [62,63,64].

Once the virus has entered the cell, the next step is to hijack the cellular machinery to translate the replicase gene, which is critical to producing non-structural proteins (NSPs). The replicase gene contains the open reading frames (ORFs) rep 1a and rep 1b, which code for the polyproteins pp1a and pp1b. These polyproteins contain the NSPs and are cleaved accordingly. These NSPs form the replicase transcriptase complex, which is necessary for RNA replication, both genomic and sub-genomic. The genomic RNA will go on to form the intra-capsid genetic material, while the sub-genomic genetic material serves as mRNA for the structural proteins. Once these proteins are translated, they migrate to the endoplasmic reticulum-Golgi intermediate compartment, where they integrate with the genetic material. Finally, the assembled virion travels to the cell membrane inside a vesicle and is released via exocytosis [39,62,63,64].

As we have thus far described, infection of the lungs and subsequent viral replication can induce lung injury in two ways, first through direct viral injury to cells in the lower airways and indirectly through an overactive immune response to this damage. Histopathological analysis revealed a prominent expression of the SARS-CoV-2 protein RP3 NP on alveolar epithelial cells, indicating that the virus is indeed present in significant quantities in the lungs [65]. The infected lungs show diffused alveolar damage in the form of denuded alveolar cells and reactive type II pneumocyte hyperplasia. Damaged epithelial cells were found in the intra-alveolar compartment as well [65,66,67]. The intra-alveolar space was also found to have extensive inflammatory infiltration, likely due to both the virus’s presence and damage to the cells of the lower airways. This is an important concept to help explain the transition from pneumonia to ARDS, a potentially lethal complication of COVID-19. Once diffuse alveolar injury develops as a result of COVID-19 pneumonia, it is a key driver of the overproduction of inflammatory cytokines, including IL-1, IL-6, IL-8, and TNFα [68,69,70,71]. This causes extensive neutrophil migration to the lungs and the subsequent release of toxic products such as reactive oxygen species and neutrophil elastase [72,73]. This causes damage to the vascular endothelium and alveolar epithelium, leading to fluid accumulation within the alveolar spaces, impairing oxygen-carbon dioxide exchange, and leading to ARDS secondary to the injury caused by COVID-19 pneumonia.

The recently published literature suggests that the respiratory system’s infected individual composition microbiome is varied from healthy persons. The normal microbiota stability might be different due to age and diet; these micro-ecosystems retain the respiratory system’s strength [24]. Infection and patient death are increasing, leading to doctors’ and researchers’ great concern because infected patient symptoms are similar to common flu. As per the latest World Health Organization (WHO) guidelines, the specimen obtained from the throat, sputum, and blood samples COVID-19 diagnosis must be checked by reverse transcription-polymerase chain reaction (rRT-PCR). These results validate conventional diagnostic techniques like a chest ultrasound and computed tomography (CT) imaging [74]. The widespread contagion COVID-19 is more concerned because of a lack of immune protection and treatment and the diagnostic tool and mass-level management of infected patients. Current medical knowledge and research on severe and critical patients management and experimental therapies are still evolving. Still, several protocols on minimizing the risk of infection among the general population, patients, and healthcare workers have been approved and diffused by International Health Authorities [6].

## 5. Future Directions for the Clinical Management of COVID-19

In the context of mild infection, which is true for the majority of cases, the CDC in the United States and health authorities of several other countries recommend home isolation with supportive measures [21,75,76]. Recovery has been outlined as a resolution of fever without antipyretic medication and improvement in cough or dyspnea along with a negative test result. In the hospital setting, clinical management again involves supportive care and meticulous infection control. According to the WHO, SARS-CoV-2 warrants contact and droplet precautions, which involves a mask, gown, and gloves. If healthcare providers are engaging in aerosol-generating procedures, such as intubation, then the recommendation is to adopt airborne precautions, which include a respirator.

To support these infection control protocols, it is critically important to have a coordinated triaging system, typically in the setting of an emergency department or an outpatient clinic [6]. Such triaging systems have already been reported in the literature and play an important role in facilitating the response to a potentially infected patient, prior to determining their disease status through testing [6]. Indeed, once patients present to medical attention, it is also imperative that their disease status be identified in an organized and timely manner, this can involve the development of rapid diagnostic kits that leverage the well-established rt-PCR test [74].

The development of hypoxia secondary to pneumonia or ARDS warrants intubation and mechanical ventilation. Hypoxia refractory to mechanical ventilation may require extracorporeal membrane oxygenation [77]. Given the baseline risk of thrombus formation in hospitalized patients and the increased risk specifically attributed to COVID-19, a number of international bodies, including the International Society of Thrombosis and Haemostasis (ISTH), recommend prophylactic doses of low-molecular-weight heparin in the absence of contraindications, such as active bleeding [78].

While no medications have definitively been shown to improve outcomes in patients with COVID-19, there are several agents currently being investigated. Remdesivir is a prodrug initially developed to treat Ebola that is metabolized to the active metabolite GS-441524, which is an adenosine analog. GS-441524 is capable of impairing viral RNA polymerase, thereby reducing viral RNA production. Recently, Remdesivir has been investigated as a potential treatment for SARS-CoV-2 because it was effective against SARS and MERS-CoV in both in vitro and in vivo studies [79,80]. A recent double-blind, randomized control trial involving 1063 patients found that Remdesivir reduces the median hospitalization day from 15 in the placebo group to 11 in the treatment group (recovery was defined as discharge from hospital or cessation of supplemental oxygen [81]. Hence, the FDA has provided emergency authorization in May 2020 to use Remdesivir to treat COVID-19. Another study involving 237 participants found a trend towards faster recovery with Remdesivir but not a statistically significant difference [82]. Though both studies evaluated treatment for 10 days, it should be noted that this study had a smaller sample size, involved the simultaneous use of other therapies such as corticosteroid and antivirals, and notably, the participants in the Remdesivir group were more likely to have comorbidities such as hypertension, diabetes mellitus, and coronary artery disease [82].

Dexamethasone, a well-known, cheap, and widely-used corticosteroid, has also been investigated in a pre-print in the United Kingdom involving 2104 that has already shifted clinical practice in several countries around the world [83]. The main metric assessed in this study was a reduction in mortality, which varied depending on the level of supplemental oxygen support required. For patients on invasive mechanical ventilation, it was found there was approximately a one-third reduction in mortality, compared to a one-fifth reduction in those who required oxygen but did not receive it through invasive ventilation [83]. Crucially, patients not receiving respiratory support did not experience a reduction in mortality. Dexamethasone is used in the treatment of severe COVID-19 but not in prophylaxis or to treat mild cases [83]. Although many countries, including the United Kingdom and the United States, now recommend the use of dexamethasone in the case of severe infection requiring ventilation, it is important to verify this data with subsequent investigations to draw more definitive conclusions.

Another agent that has been investigated in recent weeks is convalescent plasma containing antibodies against SARS-CoV-2, collected through donations from patients who have recovered from COVID-19 [84]. While convalescent plasma has not been shown to have a significant effect in a recently published study involving 103 patients, it is still being collected for use in other ongoing clinical trials [84]. It was found that at 72 h, there was a significant reduction in nasopharyngeal viral RNA clearance (87% vs. 38% in the control group), but there was not a significant improvement in more clinically relevant variables such as mortality (16% vs. 24% in the control group [84]. Further data is certainly required before more definitive conclusions can be drawn. Another potential adjunct treatment involves human recombinant soluble ACE2, which has been shown to inhibit infection and viral growth in vitro. Viral growth was shown to be inhibited by 1000 to 5000 fold in Vero cells treated with soluble ACE2 and to a significant degree in mouse models in vivo [85].

Previously, hydroxychloroquine, a well-known anti-malarial often used daily in the treatment of rheumatoid arthritis and systemic lupus erythematosus, was investigated [86]. However, the data behind this drug has been controversial, resulting in high-profile retractions. Currently, there are no high-quality data to indicate that hydroxychloroquine would be beneficial in the context of COVID-19 with the Federal Drug Administration (FDA) in the United States revoking its emergency use authorization [87]. The potential severe side effects of hydroxychloroquine, including permanent changes to vision and prolongation of the QT interval, must also be considered [88].

## 6. Vaccine Development

There is presumably no pre-existing antibody-based immunity in the population against this novel coronavirus, and everyone in the population is assumed to be susceptible. Antibodies generated in the host help to control the virus in inflamed organs and limit the spread of the virus to other organs via systemic circulation [89,90,91,92,93]. However, it is important to note that the adaptive immune system requires 2–4 weeks to produce a considerable amount of antibodies to mount a robust antibody-mediated humoral or T-cell mediated immune response. In SARS-CoV2-infected patients, a significant level of antibodies is detectable only ~10 days after the infection [94,95]. Antibodies are considered to prevent subsequent infections, and vaccines are being developed to increase an additional layer of immunity. However, there are no vaccines currently available against SARS-CoV-2 to provide this additional layer of immunity.

Thanks to the global response to the COVID-19 pandemic, there has been important progress towards developing a vaccine. On 16 March 2020, a volunteer in the United States was the first to receive a potential SARS-CoV-2 vaccine known as mRNA-1273, which was manufactured by ModernaTX, as part of a Phase I safety trial [96]. The time from sequencing the viral RNA to developing this prototype was only an unprecedented 42 days, due to the advances in nucleic acid-based vaccine strategies [97,98]. Nucleic acid-based vaccines are considered to be superior to live attenuated or subunit vaccines because they do not require developing large quantities of the pathogen in a laboratory setting, can be used more safely in immunocompromised individuals who are most at risk from COVID-19, and still induce a robust T and B cell response [99]. However, the process of developing an effective, safe, and widely available vaccine is expected to take 12 to 18 months [100,101]. On the other hand, with an unprecedented number of new investigational agencies and companies and an effort to find a therapeutic solution to the fast-spreading coronavirus pandemic, more than 140 vaccine trials are being undertaken by several countries and the WHO-coordinated SOLIDARITY trial [99,102,103,104,105,106].

The development of a vaccine against SARS-CoV-2 requires a detailed understanding of how this novel coronavirus developed, its viral structure, as well as the targets of vaccines that have been developed against related viruses, such as SARS-CoV and MERS. The information available on the zoonotic transmission of SARS-CoV-2, as well as the proteins required for viral function, facilitate epitope selection for vaccine development. It is now known that SARS-CoV-2 shares 79.5% and 96.2% of its genetic sequence with SARS-CoV and the bat coronavirus, respectively. Bat coronavirus is the origin of the current outbreak. Researchers believe that it is unlikely that the virus is transmitted directly from bats given that there is not as much similarity as expected from the previous precedent, especially in the receptor-binding domain (RBD) of the spike (S) protein [107,108]. For the context, SARS-CoV is believed to have gone through civet cats as an intermediate host, and there was a 99.8% similarity between those two viruses. There has been work investigating whether the intermediate host was the pangolin, an anteater; however, despite having a 99% at the RBD, the overall similarity was only 90.3% in the population studied [107].

With regard to vaccine development, the structural proteins have been the primary target of developing vaccines against SARS-CoV and MERS-CoV [109,110,111,112]. The N protein binds to viral RNA and is necessary for viral RNA synthesis. The M and E proteins have been noted to be important players in viral assembly, with E, in particular, is important in maintaining virulence [25,102,103,104,105]. Finally, S protein plays the most important role in viral attachment, fusion, and entry, and it serves as a target for the development of antibodies, entry inhibitors, and vaccines. The S protein contains the RBD in its S1 subunit, through which both SARS-CoV and SARS-CoV-2 enter host cells via the ACE2 receptor [113].

While there may not be a formally approved vaccine for widespread use against SARS-CoV or MERS-CoV, there have been several targets identified that may be useful in the development of a vaccine against SARS-CoV-2. The first broad category of vaccines involves inactivating SARS-CoV through formaldehyde or UV light, although this is not an attractive option because of safety risks either during production or due to incomplete inactivation [114]. There have been trials involving inactivated viruses lacking the E protein, which has been shown to remove the virulence of coronaviruses and is a potential alternative. Safer options include vaccines involving the S-protein, which in isolation is not infectious. Vaccines involving either the entire S-protein or a fragment involving just the RBD have also been tested with a better efficacy and safety profile in mice. Vaccines involving RBD may also offer some protection against related coronaviruses, given that this region is typically conserved [63,64,102,115]. Similarly, a DNA vaccine against MERS-CoV involving the full S protein was shown to generate a safe and effective response in mice similar to convalescent patients. DNA vaccines involving the N protein are not considered effective, although there is some evidence that co-administration with the M protein can augment this effect [28,109,113,114,115,116]. The use of DNA and RNA-based strategies considerably shortened the time required for many vaccine candidates to enter Phase I trials [96,102,104,105,116]. Reccent reports show that two mRNA vaccines, by Moderna and BioNTech/Pfizer, are highly effective (95%) in controlling COVID-19, in phase III trials. Hence, these vaccines are considerd by FDA for emergency use authorization [117].

## 7. Conclusions

Currently, COVID-19 presents a significant challenge to healthcare systems around the world due to several factors, including the route of transmission and infectivity, a lack of formally tested therapeutics and vaccines, and extensive individual mobility before the imposition of social distancing public health measures. Understanding the similarities and differences between SARS-CoV-2 and related coronaviruses such as SARS-CoV and MERS will raise important questions that can inform the development of novel therapeutics and vaccines. Specifically, comparing and contrasting the viral replication cycle, routes of infection, and clinical manifestations of these viruses is critical to the testing of antivirals such as remdesivir, while other therapies such as dexamethasone and convalescent and monoclonal antibodies are also being further investigated. Finally, the development of computer modeling is essential to public health planning, particularly related to social isolation and healthcare resource allocation policies. Prior coronavirus research in tandem with modern techniques may provide an effective and coordinated response to the global COVID-19 pandemic.
